# Supplementary material for: EnzML: multi-label prediction of enzyme classes using InterPro signatures
Source: BMC Bioinformatics. 2012 Apr 25;13:61. doi: 10.1186/1471-2105-13-61 (PMC3483700; doi:10.1186/1471-2105-13-61)
Supplement: Addtional file 5 — The Java code to format the data files, evaluate and predict. The file enzml_java_code.tar.gz contains the Java code used to format database data to ARFF and XML formats, to execute cross and train-test (jackknife) evaluations and to record evaluation results to database. More information is included in the readme.txt file and the Javadoc files. The code can be used with a MySQL database. To use a different database software, other JDBC drivers might be required. [file 1471-2105-13-61-S5.gz › java_code/enzml2011/doc/uk/ac/ed/inf/csb/taweka/TaWekaProperties.html]

TaWekaProperties


---


|  |  |  |
| --- | --- | --- |
| **Package** \| **Class** \| **Use** \| **Tree** \| **Deprecated** \| **Index** \| **Help** | |  |
| **PREV CLASS**   **NEXT CLASS** | **FRAMES**    **NO FRAMES**     **All Classes** |  |
| SUMMARY: NESTED \| FIELD \| CONSTR \| METHOD | DETAIL: FIELD \| CONSTR \| METHOD |  |


---


## uk.ac.ed.inf.csb.taweka Class TaWekaProperties

```
java.lang.Object
  uk.ac.ed.inf.csb.taweka.TaWekaProperties
```

---

``` public class TaWekaProperties extends java.lang.Object ```

Contains the properties for a TaWeka run
It contains:
1. the properties read from properties file
2. the path of the properties file itself
3. the name of the arff file including a timestamp

**Version:**
:   24-Mar-2007

**Author:**
:   Luna De Ferrari

---

| **Field Summary** | |
| --- | --- |
| `static java.lang.String` | `ARFF_FILE_PATH_PROP`             the file path where the ARFF file will be written, inclusive of file name. |
| `static java.lang.String` | `ATTRIBUTES_QUERY_PROP`             A list of attribute names |
| `static java.lang.String` | `ATTRIBUTES_VALUES_QUERY_PROP`             The Attributes values query. |
| `static java.lang.String` | `CLASS_NAME_PROP`             The class name. |
| `static java.lang.String` | `CLASS_VALUES_QUERY_PROP`             The Class values query. |
| `static java.lang.String` | `DATABASE_PROP`             the database name to query the data from |
| `static java.lang.String` | `DESCRIPTION_PROP` |
| `static java.lang.String` | `EXPERIMENT_PROP`             the experiment short name |
| `static java.lang.String` | `INSTANCE_CLASS_QUERY_PROP`             Instance class query: gets the class value for each instance. |
| `static java.lang.String` | `INSTANCES_QUERY_PROP`             The query to get a list of instances identifiers. |
| `static java.lang.String` | `PROPERTIES_FILE_PATH_PROP`             the path to the properties file itself, from which the other properties were read |


**Constructor Summary**

`TaWekaProperties(java.lang.String propertiesFilePath)`
  
          Constructor
loads the properties from file


| **Method Summary** | |
| --- | --- |
| `java.util.Properties` | `getProperties()`             Get the properties |
| `java.lang.String` | `getPropertiesFilePath()`             Get the properties file path |
| `static void` | `main(java.lang.String[] args)` |

**Methods inherited from class java.lang.Object**

`equals, getClass, hashCode, notify, notifyAll, toString, wait, wait, wait`

**Field Detail**

### PROPERTIES\_FILE\_PATH\_PROP

```
public static final java.lang.String PROPERTIES_FILE_PATH_PROP
```

:   the path to the properties file itself, from which the other properties were read

    **See Also:**: Constant Field Values

---


### DATABASE\_PROP

```
public static final java.lang.String DATABASE_PROP
```

:   the database name to query the data from

    **See Also:**: Constant Field Values

---


### EXPERIMENT\_PROP

```
public static final java.lang.String EXPERIMENT_PROP
```

:   the experiment short name

    **See Also:**: Constant Field Values

---


### DESCRIPTION\_PROP

```
public static final java.lang.String DESCRIPTION_PROP
```

**See Also:**: Constant Field Values

---


### ARFF\_FILE\_PATH\_PROP

```
public static final java.lang.String ARFF_FILE_PATH_PROP
```

:   the file path where the ARFF file will be written, inclusive of file name.
    A timestamp and the .arff ending will be added automatically.

    **See Also:**: Constant Field Values

---


### ATTRIBUTES\_QUERY\_PROP

```
public static final java.lang.String ATTRIBUTES_QUERY_PROP
```

:   A list of attribute names

    **See Also:**: Constant Field Values

---


### ATTRIBUTES\_VALUES\_QUERY\_PROP

```
public static final java.lang.String ATTRIBUTES_VALUES_QUERY_PROP
```

:   The Attributes values query. Using '?' as instance id generates a prepared statement:
    the '?' will be iteratively substituted with each instance id
    to get the attribute values for that instance (presence of certain attributes).
    Example: attributesValuesQuery = SELECT DISTINCT attribute FROM instanceToAttributeTable \
    WHERE instance LIKE ?
    ORDER BY attribute

    **See Also:**: Constant Field Values

---


### INSTANCE\_CLASS\_QUERY\_PROP

```
public static final java.lang.String INSTANCE_CLASS_QUERY_PROP
```

:   Instance class query: gets the class value for each instance.
    Using '?' as id generates a prepared statement.
    Example: instanceClassQuery=SELECT DISTINCT classValue FROM instanceToClassTable \
    WHERE instance LIKE ?

    **See Also:**: Constant Field Values

---


### INSTANCES\_QUERY\_PROP

```
public static final java.lang.String INSTANCES_QUERY_PROP
```

:   The query to get a list of instances identifiers.
    To be used in the classValue and attributeValues queries.

    **See Also:**: Constant Field Values

---


### CLASS\_NAME\_PROP

```
public static final java.lang.String CLASS_NAME_PROP
```

:   The class name. Examples, for a medical prediction: has\_disease.

    **See Also:**: Constant Field Values

---


### CLASS\_VALUES\_QUERY\_PROP

```
public static final java.lang.String CLASS_VALUES_QUERY_PROP
```

:   The Class values query. Gets a list of all possible values that the class can take.

    **See Also:**: Constant Field Values


**Constructor Detail**

### TaWekaProperties

```
public TaWekaProperties(java.lang.String propertiesFilePath)
```

:   Constructor
    loads the properties from file

    **Parameters:**: `propertiesFilePath` - the path to the properties file e.g. /home/me/myexperiment.props


**Method Detail**

### getProperties

```
public java.util.Properties getProperties()
```

:   Get the properties

---


### getPropertiesFilePath

```
public java.lang.String getPropertiesFilePath()
```

:   Get the properties file path

---


### main

```
public static void main(java.lang.String[] args)
```

:   **Parameters:**: `args` -


---


|  |  |  |
| --- | --- | --- |
| **Package** \| **Class** \| **Use** \| **Tree** \| **Deprecated** \| **Index** \| **Help** | |  |
| **PREV CLASS**   **NEXT CLASS** | **FRAMES**    **NO FRAMES**     **All Classes** |  |
| SUMMARY: NESTED \| FIELD \| CONSTR \| METHOD | DETAIL: FIELD \| CONSTR \| METHOD |  |


---
